# Deformation of Polyethylene Subjected to Static and Nonstatic Stresses and Krypton Ions Irradiation

**DOI:** 10.3390/polym17081081

**Published:** 2025-04-17

**Authors:** Anatoliy I. Kupchishin, Artem L. Kozlovsky, Marat N. Niyazov, Kairat B. Tlebaev, Oleksandr. V. Bondar, Alexander D. Pogrebnjak

**Affiliations:** 1 Physico-Technological Center, Abai Kazakh National Pedagogical University, Dostyk, 13, Almaty 050010, Kazakhstan; tlebaev@mail.ru; 2 Al-Farabi Kazakh National University, 71 Al-Farabi Ave., Almaty 050040, Kazakhstan; 3 Institute of Nuclear Physics, 1 Ibragimova Str., Almaty 050057, Kazakhstan; artem88sddt@mail.ru; 4 Faculty of Electronics and Information Technologies, Sumy State University, 116 Kharkivska St., 40007 Sumy, Ukraine; oleksandr.v.bondar@gmail.com (O.V.B.); a.d.pogrebnjak@gmail.com (A.D.P.); 5 Faculty of Material Science and Technology in Trnava, Institute Materials, Slovak University of Technology in Bratislava, 91724 Trnava, Slovakia

**Keywords:** polyethylene, ion irradiation, mechanical properties, krypton ions, exponential model, radiation dose

## Abstract

The dependence of polyethylene deformation on applied mechanical stress under varying load conditions and radiation doses was investigated experimentally. Obtained results reveal significant alterations in the mechanical properties of polyethylene following irradiation with krypton ions at doses of 1.5 × 10^6^, 1.6 × 10^7^, 5.0 × 10^8^, and 1.0 × 10^9^ ions/s. The stress–strain curves obtained for both the unirradiated and irradiated samples are numerically modeled using frameworks developed by the authors. The findings indicate that irradiation with krypton ions at an energy level of 147 MeV exerts a pronounced impact on the deformation and strength characteristics of polyethylene. Notably, increasing the radiation dose to 10^9^ particles/s results in a 2.5-fold increase in the rate of mechanical stress. Furthermore, the degree of deformation distortions in molecular chains induced by high-energy Kr^15+^ ion irradiation has been quantified as a function of irradiation fluence. Increasing the irradiation fluence from 10^6^ ion/cm^2^ to 10^7^ ion/cm^2^ causes only minor variations in deformation distortions, which are attributed to the localized isolation of latent tracks and associated changes in electron density. A comparative analysis of the mechanical behavior of irradiated polymer materials further revealed differences between ion and electron irradiation effects. It was observed that Teflon films lose their plasticity after irradiation, whereas polyethylene films exhibit enhanced elongation and tearing performance at higher strain values relative to their non-irradiated counterparts. This behavior was consistently observed for films irradiated with both ions and electrons. However, an important distinction was identified: high-energy electron irradiation degrades the strength of polyethylene, whereas krypton ion irradiation at 147 MeV does not result in strength reduction.

## 1. Introduction

Modern polymers used in seals, movable joints, and other engineering components must fulfill numerous performance requirements, including the ability to operate across an extensive temperature range, resistance to a variety of liquids, and durability against aging and oxidation. To meet these stringent demands, scientists have been actively developing advanced polymers and composite materials while extensively studying their structural and mechanical properties [1,2]. Strength, inherently linked to the bulk properties of materials, necessitates modifications to the material’s structure to achieve required enhancements. Ion and electron irradiation techniques have frequently been utilized in this context, enabling the improvement of mechanical characteristics, particularly the surface properties of materials, while preserving their bulk integrity.

For instance, previous studies have investigated the physical, chemical, and mechanical phenomena in irradiated materials implanted with nitrogen and helium ions at energies of 100 keV [3]. The mechanical behavior of ion-irradiated rubber was explored in [4], while the structural characteristics of pristine and irradiated polymer insulation were evaluated using scanning electron microscopy (SEM) in [5,6]. These investigations have consistently demonstrated that the structural properties of polymers are intricately linked to ion irradiation. One of the significant benefits of ion beams lies in their ability to achieve the highest energy loss density among all radiation modalities, including gamma rays, electron beams, and ion beams [7,8]. Consequently, ion irradiation is increasingly employed to enhance properties such as friction reduction, wear resistance, improved strength, and ductility, with minimal impact on bulk material characteristics—a particularly relevant approach for studying polyethylene films and their structural and mechanical properties.

Current reviews of irradiated polymers highlight the breadth of knowledge in this field and underscore the utility of ion irradiation in polymer processing [9,10]. Notably, irradiation induces critical surface modifications, such as shrinkage and smoothing of the polymer’s outer layer, processes believed to be associated with hydrogen release from the surface [11,12]. The diverse array of radiation sources used in polymer processing introduces defects into the material’s microstructure, thereby altering properties, including mechanical ones. The relationship between absorbed radiation dose and the physical and mechanical properties of conventional polymers represents a cutting-edge area of research [13,14]. Polyethylene (PE), a prominent polymer material, has increasingly replaced traditional materials in various industries, including nuclear and aerospace applications such as cables, missile shields, and gasoline pumps [9].

Studies investigating the effects of low-dose radiation (up to 200 kGy) on a variety of polymers have revealed that modifications in crystallinity and tensile strength arise from competing processes of cross-linking and chain scission events in the polymer network [11,15,16]. Researchers have also examined novel methods of altering polymer surfaces, such as applying PDA media coatings followed by the incorporation of functional groups. A combination of experimental techniques and computational modeling—most notably molecular dynamics (MD)—has been leveraged to investigate deformation mechanisms and the mechanical failure of individual chains in fluoroplastics and polyethylene [9,17]. In recent years, a set of research papers has explored the characteristics and applications of polymers and their composites across diverse fields, including construction, aerospace, and electronics [18,19]. Furthermore, several studies have focused specifically on the impacts of different types of irradiations on the mechanical properties of polymers and their composites [20,21].

Polyethylene films, for example, are widely utilized for wire insulation and hold potential for broader applications in the construction and aerospace industries. In addition to their practical uses, polyethylene films are valuable models for studying deformation properties in greater detail. Despite the wealth of research on irradiated polymers, the literature remains limited in addressing the combined effects of mechanical load and ion radiation on the mechanical properties of polyethylene. This study seeks to fill this critical gap, contributing to a deeper understanding of the interplay between these factors and their influence on the polymer’s structural and mechanical behavior.

## 2. Experimental Methods

Polyethylene film (PE) with a thickness of 23 microns was selected as the test material. The equipment for producing this polymer is a high-pressure autoclave made of high-strength steel that can withstand pressures up to 100 atmospheres. The installation is equipped with an anchor stirrer and jacket for heating and cooling. At the preparatory stage, the autoclave was cooled, and air was removed. The temperature was adjusted to between 70 and 80 °C, and the pressure ranged between 40 and 100 atm. The reaction ended when the pressure dropped, indicating the completion of the material manufacturing process. The product yield in one hour was 80% of the total raw material mass.

The film sheets were cut into strips using a special device with an adjustable width. The width of the strips was 5 mm, and the length of the working samples was 5, 7, 10, and 12 cm. The film samples obtained were irradiated with different doses of 1.5 × 10^6^, 1.6 × 10^7^, 5.0 × 10^8^, and 1.0 × 10^9^ ions/s with krypton ions having an energy of 147 MeV, under environmental conditions. The film samples made of Teflon were irradiated with a dose of 5 kGy in ambient conditions using high-energy electrons with an energy of 2 MeV. The third type of film studied was polyethylene, which was irradiated with doses of 100 and 200 kGy in ambient conditions using high-energy electrons with an energy of 2 MeV. Some samples were not exposed to radiation and served as control samples. The film samples for irradiation were placed at 300 mm from the accelerator’s exit window, and the beam current was 0.16 μA/cm^2^. The dose distribution inside the accelerator room was determined using the DRG-01t1 dosimetric system. The temperature of the material during the study was 23 °C.

In addition, tensile tests were conducted following the ASTM-D882 standard [22] using a universal testing machine, model RU-50. The tests were performed at a stroke speed of 16 mm/min and an alternating current frequency of 10 Hz, which was controlled by a CHNT inverter. To research the relationship between elongation and stress (mechanical stress, *σ*) and other relationships, we have upgraded our equipment. It now allows us to measure parameters using motion and force sensors under different loads and observe their changes over time. The installation uses an interface with motion and force sensors from Science Cube. The frequency of strain data collection is 2.5 mm·s^−1^, and the measurement of mechanical stress differs due to the change in Young’s modulus during elongation.

## 3. Research Results

**Figure 1:**
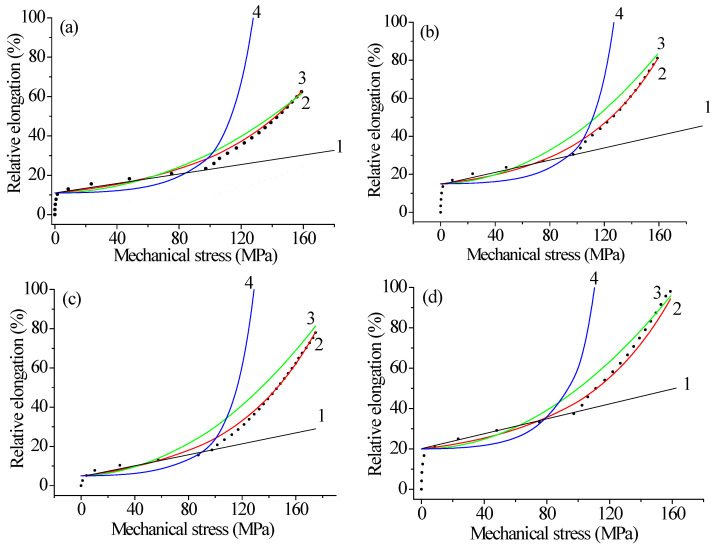
Dependence of the relative elongation on the mechanical stress of a 5 cm polyethylene film unirradiated and irradiated with 147 MeM krypton ions. Curve 1—linear; 2—exponential; 3—parabolic; 4—quadratic-exponential model; points—experimental results. (**a**)—unirradiated sample; (**b**)—irradiated with a dose of 1.5 × 10^6^ ions/s; (**c**)—1.6 × 10^7^ ions/s; (**d**)—1.0 × 10^8^ ions/s.

The results of the tensile tests using the RU-50 bursting machine on irradiated and unirradiated samples are presented in Figure 1 as graphs of the deformation versus mechanical stress. It follows from the results that, with increasing stress, the deformation of materials in the elastic region first increases linearly (as described by Hooke’s law) and then sharply increases exponentially [23]. After irradiation with krypton ions, the material samples become more ductile and begin to fracture at higher deformations than before irradiation [24]. At the same time, there is an increase in relative elongation compared to unirradiated material, and the tensile strength of the material increases for samples of all lengths.

At the same time, there is an increase in the relative elongation compared to the unirradiated material. The tensile strength of the material is also increased for all sample lengths. The effect of the flow of charged krypton ions on the material is similar in many ways to that of an electron beam [25]. The change in deformation and strength characteristics is due to the ongoing destruction of the material’s structure.

Visual inspection with the naked eye and an optical microscope did not reveal any changes in optical properties. This may be due to the fact that radiation-chemical processes have a high impact not on the surface of the polymer but on its macromolecules located on the surface. Also, the high energy of the particles allows them to penetrate through the polymer without causing significant surface defects. As can be seen from Figure 1, the deformation of the polymer increased by more than three times, and the strength increased by two times (Figure 1b). One possible explanation for these changes is the straightening of the chains of polyethylene macromolecules. Due to the large number of atoms in their structure, these chains cause such deformation and strength as they interact with each other and attempt to return the sample to its original shape even after exceeding the elastic limit.

The elastic limit of the material ends at 5–15%. More precisely, for each experiment, this can be seen in Figure 1. The curves are well described by an exponential model. The formula for this model was obtained using the cascade probabilistic method (CPM).

(1) $$\varepsilon =\varepsilon_{1}+\varepsilon_{0}(exp(\sigma /\sigma_{0})-1)$$

**Figure 2:**
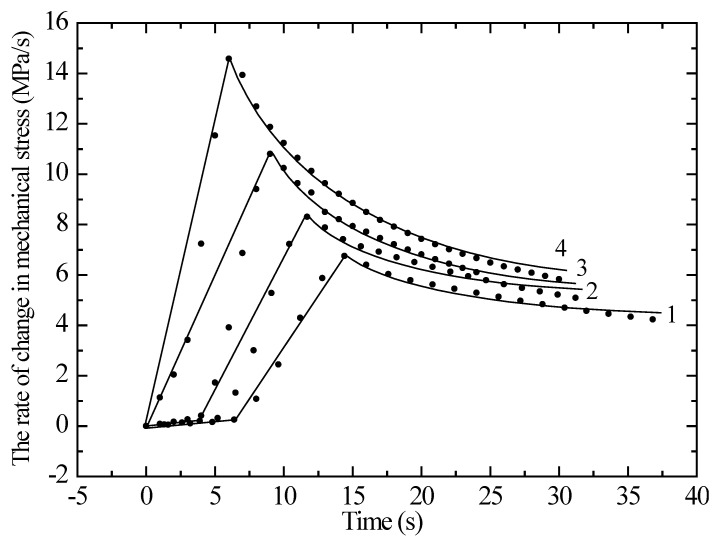
Dependence of the rate of mechanical stress on the time for an unirradiated and irradiated krypton ion polyethylene film with a working length of 5 cm. Curve 1—unirradiated sample; 2—1.5 × 10^6^; 3—1.6 × 10^7^; 4—5.0 × 10^7^ ions/s.

In Equation (1), *ε*_0_ is the limit value of the elongation, and *σ*_0_ is the mechanical stress at which the parameter (*ε*/*ε₀* − 1) decreases by a factor of *e*. Figure 2 shows graphs of the dependence of the mechanical stress rate on the control time and irradiated samples with a working length of 5 cm. From the figure, it can be seen that the first half corresponds to a linear model and the second half to an exponential model. The curves are well described by the models proposed by the authors of [9]. An increase in the radiation dose leads to a 2.5-fold increase in the rate of mechanical stress, followed by a sharp decline. This is due to the unwinding and straightening of macromolecular chains at the beginning of exposure of samples to radiation. During irradiation, radicals are formed in polyethylene molecules, which can interact with each other, forming new chemical bonds between the molecules; that is, cross-linking occurs.

**Figure 3:**
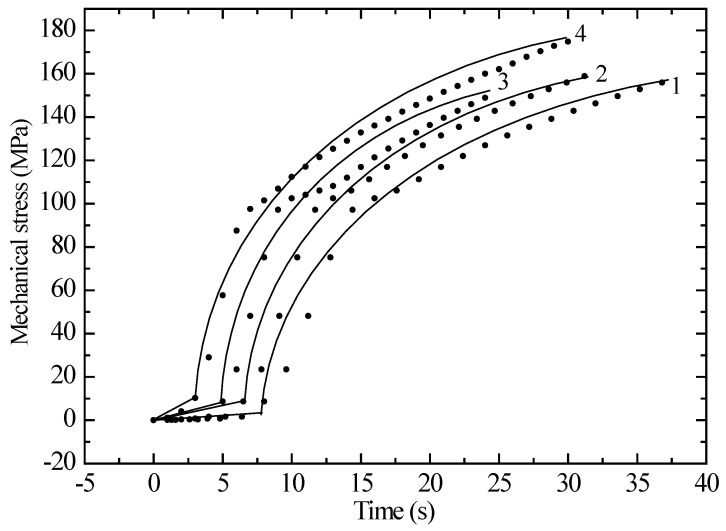
Dependence of the mechanical stress on the time for an unirradiated and irradiated krypton ion polyethylene film with a working length of 5 cm. Curve 1—unirradiated sample; 2—1.5 × 10^6^; 3—1.6 × 10^7^; 4—5.0 × 10^7^ ions/s.

**Figure 4:**
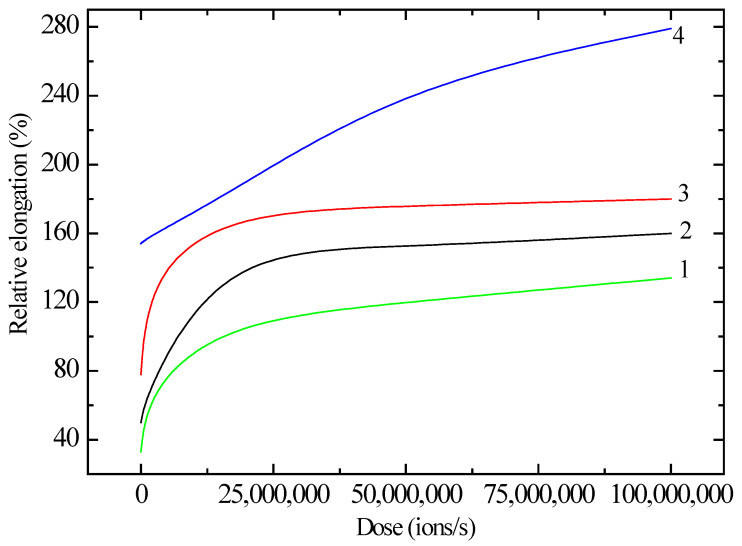
Dependence of the relative elongation on the radiation dose for polyethylene films of different working lengths. Curve 1—5; 2—7; 3—10; 4—12 cm.

However, the change in mechanical stress over time occurs according to a different scenario, as shown in Figure 3. The samples experience a significant increase in stress during the first 15 s, after which it gradually reaches saturation. This may be due to an increase in the distance between atoms in the irradiated structure of the sample. Figure 4 illustrates how the deformation of polyethylene (PE) samples of different lengths and radiation doses varies after exposure to krypton ions. Although the overall pattern of PE behavior remains unchanged after irradiation, the deformation and strength characteristics do change. As can be seen from Figure 4, the relative elongation tends to increase with increasing radiation dose. Additionally, for a sample with a working length of 12 cm (curve 4 in Figure 4), the trend of deformation appears to be different.

An increase in the deformation is accompanied by an improvement in the flexibility of the films, which is due to the continuous destruction of the polymer structure during the irradiation process.

**Figure 5:**
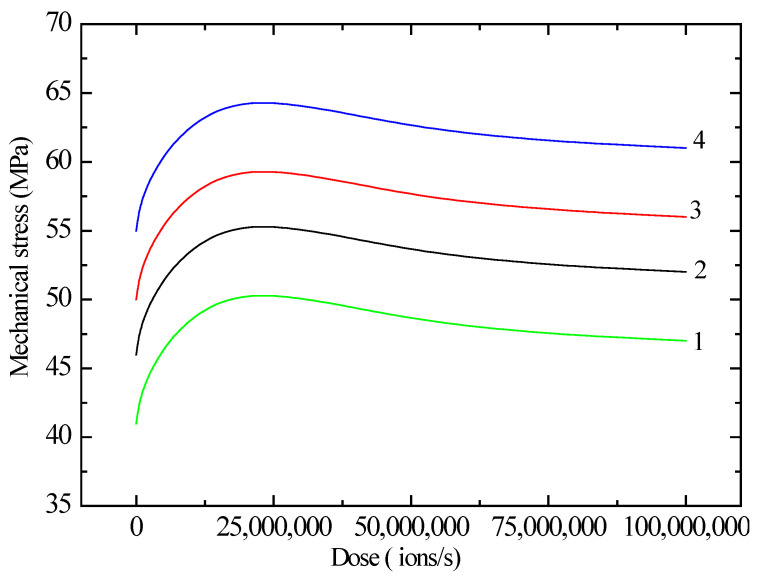
Dependence of mechanical stress on the radiation dose of a polyethylene film of different working lengths. Curve 1—5; 2—7; 3—10; 4—12 cm.

Figure 5 illustrates the relationship between mechanical stress and radiation dose for polyethylene films of different working lengths. Based on the change in the spacing between atoms and molecules resulting from stretching, we can conclude that strength increases and the forces between macromolecules increase. These findings suggest that irradiation disturbs the perfection of crystalline regions in PE, hindering the development of sliding. This causes a change in the component of inelastic deformation for plastic polyethylene after irradiation and thereby leads to a noticeable change in its mechanical properties. Further irradiation of the material causes the strength and deformation properties to deteriorate due to the fact that during the radiation process, molecules adjust to each other, and long-range order gives way to short-range order, destroying small crystallites and leading to a decrease in crystallinity and elasticity.

**Figure 6:**
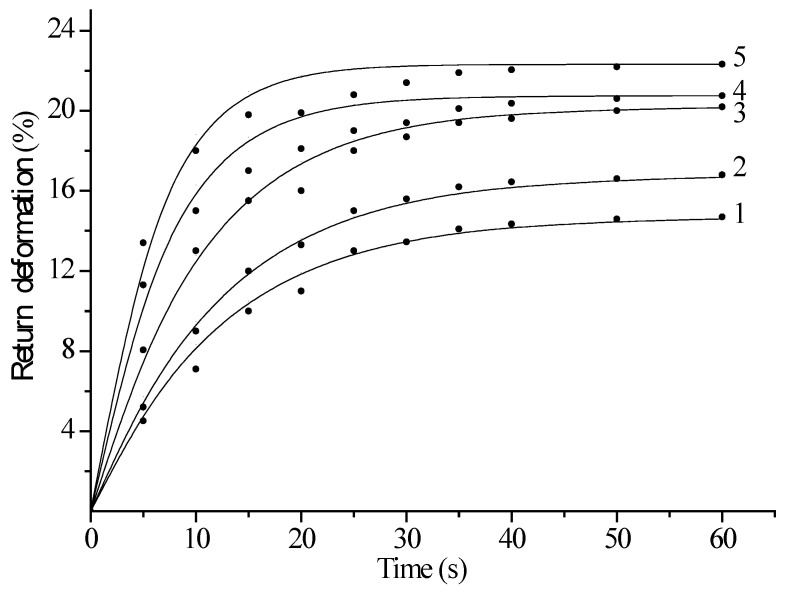
Dependence of the return deformation on time for an unirradiated and irradiated krypton ion polyethylene film with a working length of 5 cm at a static voltage of 80 MPa. Curve 1—unirradiated sample; curves 2—5 correspond to samples irradiated with doses of 2—1.5 × 10^6^; 3—1.6 × 10^7^; 4—5.0 × 10^7^; and 5—10^9^ ions/s.

Figure 6 shows the relationship between the return deformation and time for both unirradiated and krypton-ion-irradiated polyethylene films. The experiments were conducted at a static pressure of 80 MPa. As shown in the figure, the return deformation increased rapidly during the first 20 s of material loading, and then it gradually slowed down and stopped. This is because the samples have not fully lost their elasticity, and the macromolecules are attempting to restore their original shape due to interaction forces. An increase of more than two times in the return deformation after irradiation was observed, indicating an increase in attraction forces and possible partial crosslinking of macromolecules in the irradiated samples.

**Figure 7:**
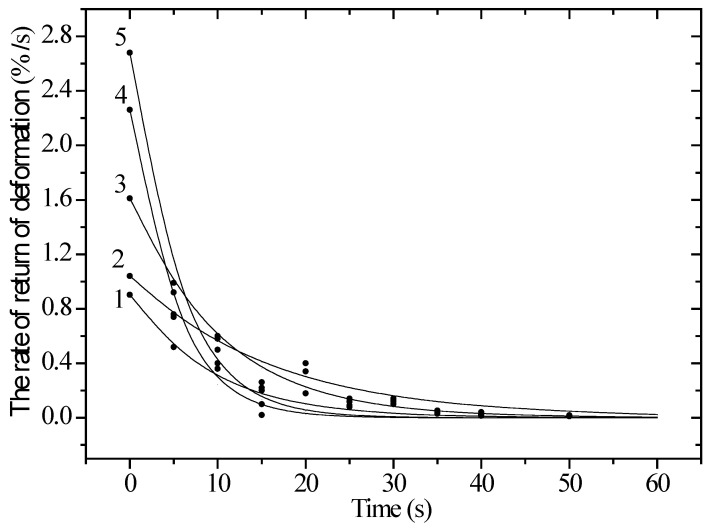
Rate of deformation recovery dependence on time for an unirradiated and krypton-ion-irradiated polyethylene film with a working length of 5 cm and a static voltage of 80 MPa. 1—unirradiated sample; 2—1.5 × 10^6^; 3—1.6 × 10^7^; 4—5.0 × 10^7^; 5—10^9^ ions/s.

It can be seen in Figure 7 that the rate of recovery deformation significantly depends on both time, static stress, and the dose of electron irradiation. Under constant load, the rate of recovery decreases with time. At a voltage of 80 MPa, the value reaches about 3% per second, due to the straightening of randomly arranged circuits in the irradiated sample. After that, the velocity decreases due to the material’s resistance to deformation, which is caused by a decrease in the cross-sectional area of the sample. This leads to an increase in the stiffness of the material. Ion irradiation of the material significantly reduces the rate of recovery deformation. This is because it decreases the frictional properties between macromolecules, changing the structure and leading to weaker straightening of polymer chains and poor sliding. Figure 1 shows the X-ray diffraction patterns of PET in its initial state and after irradiation with heavy Kr^15+^ ions at an energy of 147 MeV, as the irradiation fluence varies. The overall appearance of the diffraction patterns indicates a polycrystalline structure for the films, with a broad peak at 2θ = 23–30° and a maximum at 2θ = 25.9°. According to the assessment, the degree of crystallinity in the initial film is 53–54%, indicating a polycrystalline polymer structure. For irradiated film samples, the main changes are associated with a decrease in the intensity of diffraction peaks due to the accumulation of disordered regions caused by the deformation of molecular chains. This is also accompanied by a change in the electron density along the path of ions in the material. Figure 6 illustrates the results of measuring the magnitude of deformation distortions in molecular chains caused by irradiation with heavy Kr^15+^ ions, depending on the fluence of the irradiation.

**Figure 8:**
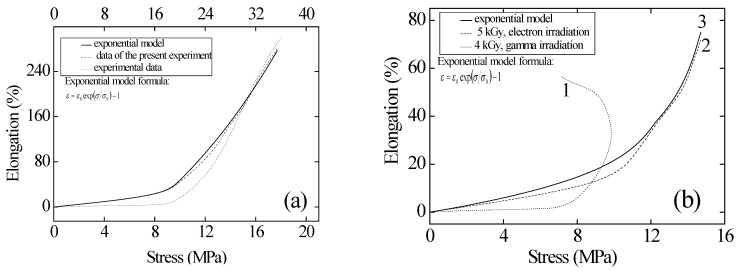
Dependence of relative elongation on mechanical stress for non-irradiated (**a**) and irradiated (**b**) PTFE films, comparison with the results of work [10].

The results of tensile tests on irradiated and non-irradiated samples, as well as their comparison with the data from [10], are shown in Figure 8. The tensile strength of the non-irradiated sample was 34 MPa. From Figure 8, it can be seen that as the stress increases, the strain in the elastic region of the materials first increases slowly, then rises sharply following an exponential law. After irradiation with electrons and gamma radiation, the material samples lose their plasticity and begin to break at lower strain than before irradiation. A significant reduction in relative elongation is observed compared to the non-irradiated material.

**Figure 9:**
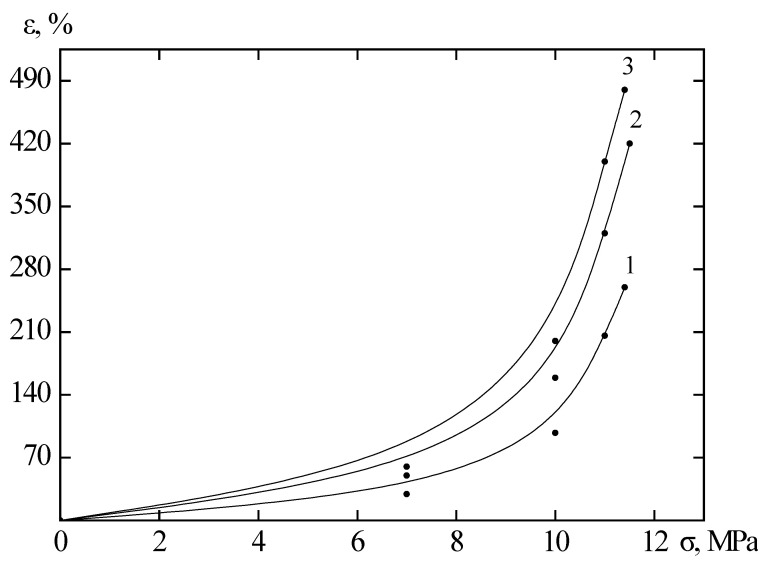
Dependence of strain on mechanical stress for polyethylene film irradiated with 2 MeV energy electrons in air. Curve 1—non-irradiated material; 2—irradiated with a dose of 100 kGy; 3—irradiated with a dose of 200 kGy.

Figure 9 shows the dependence of strain on mechanical stress for non-irradiated and irradiated polyethylene. After irradiation with doses of 100 and 200 kGy, the deformation properties improve by more than 1.5 times, and the samples begin to break at lower mechanical stress than before irradiation. A comparison of the changes in the mechanical properties of polymer materials after electron and ion irradiation, the results of which are shown in Figure 1, Figure 2, Figure 3, Figure 4, Figure 5, Figure 6, Figure 7, Figure 8 and Figure 9, led to the conclusion that Teflon films lose their plasticity after irradiation, while irradiated polyethylene films stretch better and break at higher deformation values than non-irradiated ones. This pattern is observed for films irradiated with both ions and electrons. However, after irradiation with high-energy electrons, polyethylene loses its strength, while after irradiation with krypton ions at 147 MeV, the strength does not degrade. This leads to the conclusion that krypton ions are more suitable for irradiation.

**Figure 10:**
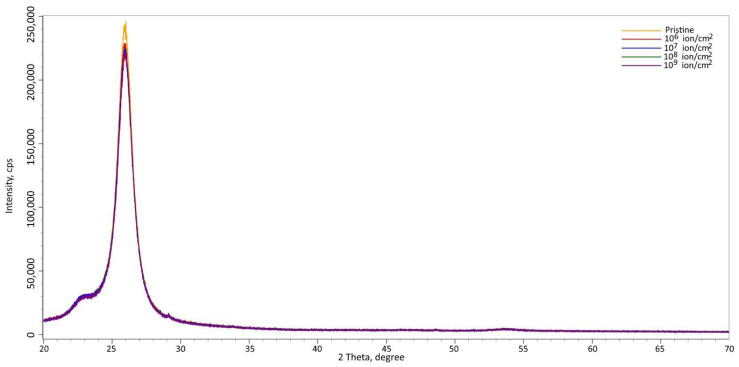
XRD patterns of PET films for different dose irradiation from 10^6^ ion/cm^2^ to 10^7^ ion/cm^2^.

The degree of deformation distortion was estimated based on the shifts in the diffraction maximum to the region of small angles shown in Figure 10. This indicates the formation of tensile stress in the polymer structure. An increase in the fluence of irradiation of a polymer film from 10^6^ ion/cm^2^ to 10^7^ ion/cm^2^ leads to small changes in the magnitude of deformation distortion caused by irradiation. This can be explained by the effect of isolated tracks and associated changes in electron density. An increase in the irradiation fluence above 10^8^ ions/cm^2^ leads to a sudden change in the pattern of deformation distortions. This is due to the convergence of structurally altered regions, or “latent tracks”, and the overlapping of their edges, which causes an increase in deformation distortion of molecular targets. This effect is associated with the anisotropic distortion of electron densities within latent tracks.

**Figure 11:**
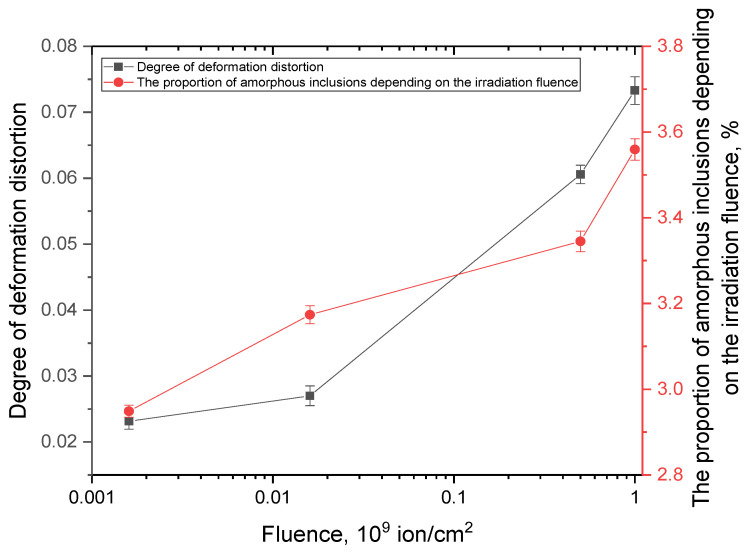
Results of the evaluation of changes in the degree of structural deformation and concentration of amorphous inclusions in the polymer due to irradiation, depending on the fluence of irradiation.

According to the data on changes in the concentration of amorphous inclusions caused by irradiation-induced changes, the increase in the proportion of these inclusions shows a clearly similar trend to that of deformation distortions as shown in Figure 11. This indicates a direct relationship between these inclusions and the structural changes in the molecular structure of the polymer resulting from irradiation.

**Figure 12:**
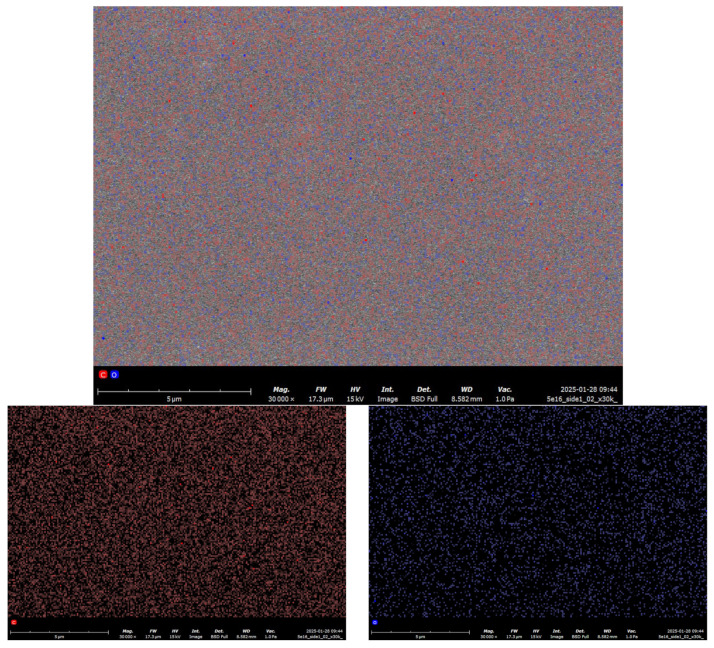
SEM-EDS C and O mapping of surface thick polyethylene film after irradiation with 147 MeV krypton ions (dose of 4 × 10^7^ particles/s) (magnification ×30,000). Red dots represent carbon; blue dots represent oxygen.

Figure 12: The images show a map of the elemental composition of polyethylene (red points C, blue points O). Below is the energy spectrum of the elemental composition obtained using EDS (SEM). Also shown is a table with a quantitative analysis of polyethylene irradiated with krypton. As can be seen from the results of the energy dispersive analysis of Figure 12 .a.b.s. samples of polyethylene irradiated with krypton, oxygen, and carbon are uniformly distributed in the surface layer. The table shows the quantitative elemental analysis, from which it is evident that the concentration ratio does not change: carbon (C) is approximately 72 atomic percent and oxygen is 28%. It is evident that the stoichiometry is not observed: three carbon atoms per one oxygen atom. In addition, it is noteworthy that irradiation at such doses does not lead to the formation of small pores, while the atoms are uniformly distributed near the surface of the polyethylene.

**Figure 13:**
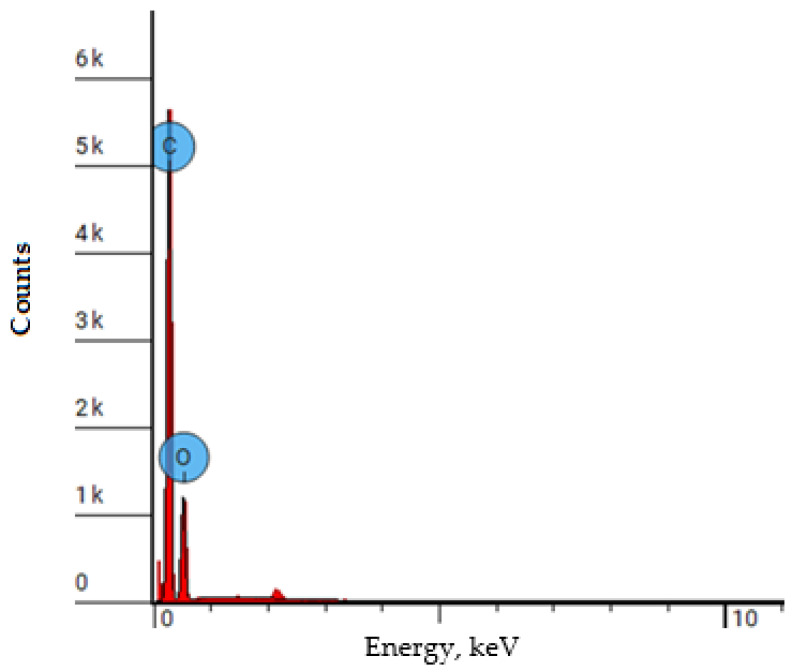
The energy dispersion X-ray analysis of polyethylene film samples with a thickness of 23 microns irradiated with krypton ions with an energy of 147 MeV with an absorption dose of 4 × 10^7^ ions/s.

The EDS shows (Figure 13) that the concentration of carbon atoms is 4 times higher than the concentration of oxygen atoms and accounts for more than 72% of the total mass, which is confirmed by Table 1.

## 4. Conclusions

1. Tensile tests conducted on irradiated and unirradiated polyethylene (PE) samples demonstrated a clear relationship between deformation and mechanical stress. Within the elastic range, deformation increases linearly following Hooke’s law before transitioning to exponential growth as stress rises. Irradiation significantly influences the deformation and strength properties of polyethylene.
2. Plots illustrating the rate of mechanical stress over time for control and irradiated samples (with a working length of 5 cm) aligned well with the predictive models proposed in this study. A notable finding was the 2.5-fold increase in the mechanical stress rate with higher radiation doses.
3. The temporal evolution of mechanical stress revealed an intense increase during the first 15 s of loading, followed by saturation. This behavior is likely due to atomic-scale structural changes in the irradiated material, such as increased interatomic distances.
4. When examining deformation under various radiation doses, the qualitative stress–strain behavior of polyethylene remained consistent; however, significant changes were observed in the material’s deformation and strength characteristics.
5. The relationship between mechanical stress and irradiation dose suggests that stretching increases interatomic and intermolecular distances, enhancing the strength of the polymer and promoting stronger intermolecular forces.
6. Recovery deformation increased rapidly during the first 20 s of loading before decelerating and halting. This is attributed to the partial retention of elasticity in irradiated samples, where macromolecules strive to revert to their original configuration due to molecular interactions.
7. The recovery deformation rate depends on time, static stress, and radiation dose. Ion irradiation reduced the recovery deformation rate, indicating significant changes to the materials’ viscoelastic behavior.
8. Deformation distortions in molecular chains caused by irradiation with heavy Kr15+ ions were evaluated as a function of irradiation fluence. A slight increase in deformation distortion was observed when the fluence increased from 10^6^ ions/cm^2^ to 10^7^ ions/cm^2^, likely due to the isolation of latent tracks and corresponding changes in electron density.
9. Comparative analysis of electron- and ion-irradiated polymer materials showed that Teflon films lose plasticity post-irradiation, whereas polyethylene films exhibit enhanced elongation and failure at higher deformation values compared to unirradiated samples. High-energy electron irradiation degrades polyethylene strength, while krypton ion irradiation at 147 MeV maintains mechanical strength, underscoring the suitability of krypton ions for irradiation processes.
10. The findings highlight the potential of irradiated polyethylene films for practical applications in wire insulation, construction materials, and the aerospace industry. Additionally, these results contribute to a deeper understanding of deformation mechanisms in irradiated polymers, paving the way for advanced material design in high-performance environments.

## Figures and Tables

**Table 1:** Data on the concentration of elements of a polyethylene film with a thickness of 23 microns irradiated with krypton ions with an energy of 147 MeV with an absorption dose of 4 × 10^7^ particles/s, obtained by elemental analysis.

Element Number	Element Symbol	Element Name	Atomic Conc.	Weight Conc.
6	C	Carbon	72.025	65.900
8	O	Oxygen	27.975	34.100

## Data Availability

The original contributions presented in this study are included in the article. Further inquiries can be directed to the corresponding authors.
